# Exploring the Role of MMP-9 and MMP-9/TIMP-1 Ratio in Subacute Stroke Recovery: A Prospective Observational Study

**DOI:** 10.3390/ijms25115745

**Published:** 2024-05-25

**Authors:** Lidia Włodarczyk, Natalia Cichon, Michał Seweryn Karbownik, Joanna Saluk, Elzbieta Miller

**Affiliations:** 1 Department of Neurological Rehabilitation, Medical University of Lodz, Milionowa 14, 93-113 Lodz, Poland; lidia.wlodarczyk@umed.lodz.pl (L.W.); elzbieta.dorota.miller@umed.lodz.pl (E.M.); 2 Biohazard Prevention Centre, Faculty of Biology and Environmental Protection, University of Lodz, Pomorska 141/143, 90-236 Lodz, Poland; 3 Department of Pharmacology and Toxicology, Medical University of Lodz, Żeligowskiego 7/9, 90-752 Lodz, Poland; michal.karbownik@umed.lodz.pl; 4 Department of General Biochemistry, Faculty of Biology and Environmental Protection, University of Lodz, Pomorska 141/143, 90-236 Lodz, Poland; joanna.saluk@biol.uni.lodz.pl

**Keywords:** post-acute stroke, MMP9, MMP9/TIMP1, neuroplasticity, rehabilitation, post-stroke depression

## Abstract

Despite the significant changes that unfold during the subacute phase of stroke, few studies have examined recovery abilities during this critical period. As neuroinflammation subsides and tissue degradation diminishes, the processes of neuroplasticity and angiogenesis intensify. An important factor in brain physiology and pathology, particularly neuroplasticity, is matrix metalloproteinase 9 (MMP-9). Its activity is modulated by tissue inhibitors of metalloproteinases (TIMPs), which impede substrate binding and activity by binding to its active sites. Notably, TIMP-1 specifically targets MMP-9 among other matrix metalloproteinases (MMPs). Our present study examines whether MMP-9 may play a beneficial role in psychological functions, particularly in alleviating depressive symptoms and enhancing specific cognitive domains, such as calculation. It appears that improvements in depressive symptoms during rehabilitation were notably linked with baseline MMP-9 plasma levels (r = −0.36, *p* = 0.025), and particularly so with the ratio of MMP-9 to TIMP-1, indicative of active MMP-9 (r = −0.42, *p* = 0.008). Furthermore, our findings support previous research demonstrating an inverse relationship between pre-rehabilitation MMP-9 serum levels and post-rehabilitation motor function. Crucially, our study emphasizes a positive correlation between cognition and motor function, highlighting the necessity of integrating both aspects into rehabilitation planning. These findings demonstrate the potential utility of MMP-9 as a prognostic biomarker for delineating recovery trajectories and guiding personalized treatment strategies for stroke patients during the subacute phase.

## 1. Introduction

According to the 2024 Heart Disease and Stroke Statistics, the age-adjusted stroke death rate in the United States increased by 8.4%, whereas the actual number of stroke deaths increased by 26.3% [1]. This suggests that despite effective preventive therapies, stroke incidence is likely to remain high due to inter alia increasing life expectancy and the prevalence of risk factors associated with chronic diseases. Moreover, stroke continues to be the primary cause of disability among individuals aged 40 and above who are actively engaged in the workforce [2]. This epidemiological data is driving the search for more effective therapeutic methods and highlights the need to understand the factors influencing treatment outcomes. Over the past decades, significant advances have been made in early treatment strategies, including intravenous tissue plasminogen activator (tPA) and endovascular thrombectomy (EVT) [3]. Indeed, evaluating prognosis and determining rehabilitation needs for post-stroke recovery can pose significant challenges for clinicians. This complexity arises from the multitude of factors influencing recovery, including both spontaneous and therapeutic-induced processes. Consequently, there is a crucial need to identify biomarkers that are capable of estimating brain recovery potential and that can serve as prognostic tools, monitor treatment response, and act as predictive tools [4].

Previous research has evaluated the potential of vascular endothelial growth factor (VEGF), insulin-like growth factor 1 (IGF-1), and matrix metalloproteinase 9 (MMP-9) protein and gene expression in predicting stroke outcomes and correlated the findings with clinical assessments of cognitive function and depressive symptoms [5]. The results indicate that MMP-9 offers particular promise as a biomarker of post-stroke cognitive recovery: MMP-9 level in plasma decreased during three-week rehabilitation, with the changes correlating with improvements in cognitive function. The present study follows up on these findings by examining the correlation between MMP-9 and functional status in stroke patients.

As a zinc-dependent endopeptidase, MMP-9 plays a crucial and distinct role in both brain physiology and pathology. It is released in the brain by various cell types, including neurons, glia, and leukocytes, but its activation is localized and subject to temporal constraints. While MMP-9 is typically present at low levels in the brain, its enzymatic activity, protein level, and gene expression can be significantly upregulated in response to various physiological stimuli and pathological insults [6]. Neuronal MMP-9 participates in synaptic plasticity by controlling the shape of dendritic spines and the function of excitatory synapses [7,8]. Its level also appears to increase following the induction of late-phase long-term potentiation (LTP), which entails the enduring enhancement of synaptic strength in the CA1 region of the hippocampus [9].

Hence, it would seem that MMP-9 plays a pivotal role in learning, memory, and cortical plasticity. However, when dysregulated, MMP-9 can contribute to a wide range of brain disorders. Studies have failed to confirm that MMP-9 has any beneficial impact in the acute phase of stroke, where it primarily contributes to processes such as blood–brain barrier damage; indeed, it may serve as a prognostic biomarker of poor outcome. Post-stroke patients exhibit significantly higher concentrations of MMP-9 compared to healthy volunteers [10], and it has been found to have predictive value for hemorrhagic transformation [11] and for post-stroke cognitive impairment [12]. In addition, a growing body of evidence indicates a correlation of MMP-9 with cognitive dysfunction in both vascular dementia due to cerebral small vessel disease [13] and neurodegenerative dementia in the course of Alzheimer’s disease [14]; it has also been found to influence vascular contributions to dementia and Alzheimer’s disease. However, it is important to consider that MMP-9 also plays an important role in synaptic plasticity, and further investigation into this promising yet challenging aspect is warranted.

Matrix metalloproteinase activity is controlled by the activation of various proenzymes. In particular, their activity is effectively inhibited by tissue inhibitor of metaloproteinase (TIMPs) [15]. These bind to the active center of the MMP, containing a zinc ion, to form MMP–TIMP complexes in a 1:1 ratio, thus preventing the MMP from attaching to substrates [16,17]. Therefore, by ascertaining the MMP-9/TIMP-1 ratio, it is possible to determine the level of MMP-9 blockage or activation.

Hence, the aim of this study was to assess the prognostic value of MMP-9, TIMP-1, and MMP-9/TIMP-1 ratio, and of the expression of their genes, as markers of recovery in stroke patients. Biochemical data was acquired (MMP-9, TIMP-1, and MMP-9/TIMP-1 level) and compared with patient demographics and selected clinical scales, including orientation to time and place, registration, attention and calculation, recall, language skills, repetition, and comprehension of complex commands. The findings were also compared with functional scales measuring independence in activities and motor outcomes. The study was conducted during the early subacute phase of stroke, spanning from seven days to three months, in accordance with the Consensus Statements from the Stroke Recovery and Rehabilitation Roundtable [18].

## 2. Results

**Figure 1:**
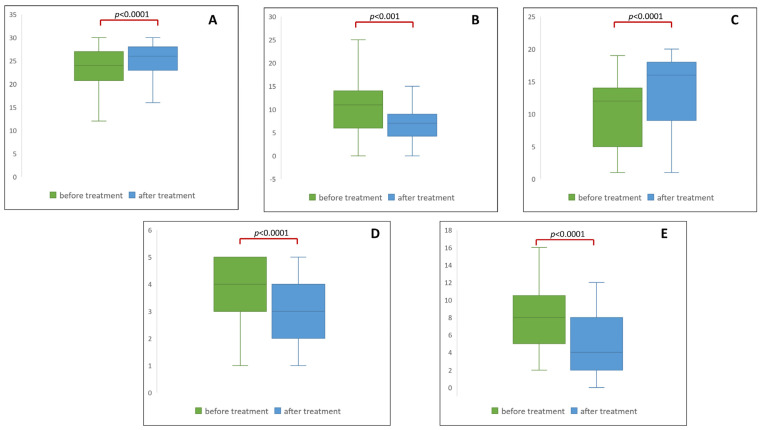
Enhancement of clinical parameters throughout post-stroke rehabilitation: (**A**) cognition expressed by the MMSE scale; (**B**) depression expressed by the GDS scale; (**C**) motor function expressed by the ADL scale; (**D**) motor function expressed by the Rankin scale; (**E**) NIHSS scale.

Intensive post-stroke rehabilitation has had a notable impact on the clinical functions of patients. Significant improvements in both the physical (assessed by ADL, Rankin, and NIHSS scales) and psychological condition (expressed by MMSE and GDS scales) were noted after the three-week rehabilitation program compared to their condition prior to rehabilitation. Further specifics are outlined in Figure 1.

**Figure 2:**
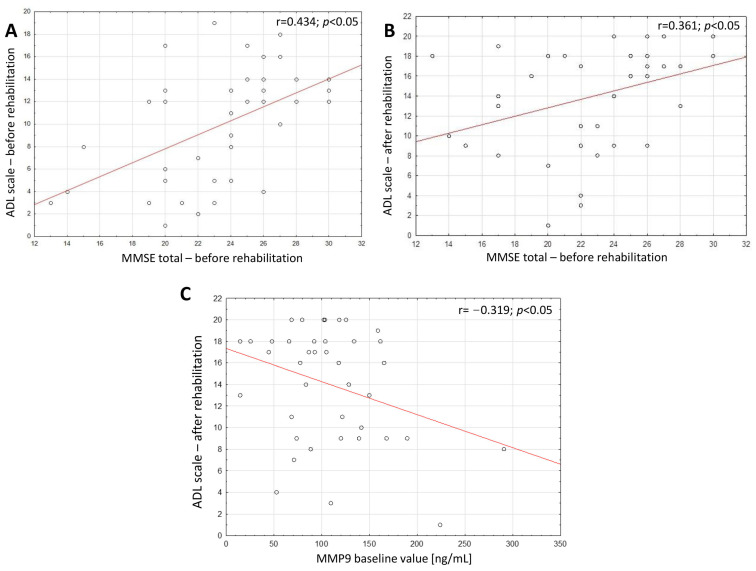
Chart illustrating the distribution of the relationship between (**A**) the baseline total MMSE score and baseline ADL scale value; (**B**) the baseline values of MMSE total and ADL scale after treatment; (**C**) the baseline values of MPP9 and ADL scale after treatment.

Furthermore, significant positive correlations were found between the initial cognitive function scores, as measured by the MMSE scale, and the motor function scores, evaluated by the ADL scale, both before (*p* = 0.005) and after treatment (*p* = 0.022). Additionally, motor function after treatment, as expressed by the ADL scale, exhibited a negative correlation with total MMP9 value at baseline (Figure 2).

No significant association was found between total MMSE score and the levels of MMP9 or TIMP1, or their ratio; however, some relationships were detected regarding MMSE subscales and these biochemical parameters. In particular, initial registration was found to be inversely proportional to the initial values of total MMP9 and TIMP1. Similarly, the change in its value correlates negatively with that of active MMP9, as indicated by the MMP9/TIMP1 ratio. Moreover, positive correlations were observed between the levels of total MMP9 and TIMP1, and their association with the attention and numeracy values recorded post-therapy. Significant negative correlations were also identified between the levels of total MMP9 and its active form (MMP9/TIMP1), and language functions (Table 1 and Supplementary Material Table S1).

The study also examined whether the associations between the studied biochemical parameters and cognitive function subdomains are linked to ischemia in the right or left cerebral hemisphere, specifically with left- or right-sided paresis. Among patients with left-sided paresis, changes in the attention and calculation domain during rehabilitation correlated positively with changes in total MMP9 and the active form (MMP/TIMP1). Further specifics regarding these associations are provided in Table 2 and Supplementary Material Table S2.

Conversely, in patients with both right- and left-sided paresis, a negative correlation was observed between total MMP9 level and language functions subscale score. Interestingly, a negative correlation was found with the active form of MMP9 (MMP9/TIMP1), but only in the subgroup with left-sided stroke. More detail is given in Table 3 and Supplementary Material Table S3.

**Figure 3:**
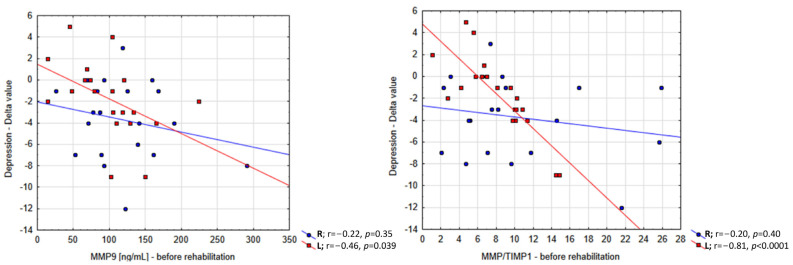
The distribution of the relationship between the enhancement of psychological function, as indicated by the GDS scale, and the baseline values of total MMP9 and its active form, represented as MMP9/TIMP1.

Improvement in depressive symptoms was predicted by background levels of MMP9 in plasma (r = −0.36, *p* = 0.025), and more substantially, by the ratio of background levels of MMP9 and TIMP1 (r = −0.42, *p* = 0.008) (Figure 3). Interestingly, the latter relationship was noted in patients with left-sided paresis (r = −0.81, *p* < 0.0001), but not with right-sided (r = −0.20, *p* = 0.40); in addition, a significant interaction was found between the side of paresis and the predictor (*p* = 0.0028). Importantly, these associations remained significant following adjustment for potential confounders such as age and sex (r = −0.87, *p* < 0.0001 for left-sided paresis; *p* = 0.029 for interaction).

## 3. Discussion

A key finding of our study is that cognition, assessed both before and after treatment, appears to be significantly positively correlated with motor function. This finding underscores the value of the relationship between cognitive and motor abilities in stroke recovery. Our findings also corroborate prior research indicating an inverse relationship between pre-rehabilitation MMP-9 serum levels and post-rehabilitation motor function scores, as measured by the ADL scale. Additionally, a notable association was found between improvements in depressive symptoms during rehabilitation and baseline MMP-9 plasma levels; however, these improvements were more closely correlated with MMP-9/TIMP-1 ratio, thus highlighting the potential role of active MMP-9 in influencing depressive symptoms.

Although motor and cognitive impairments are frequently observed following stroke, they are often managed as distinct entities, with limited evidence of their association. However, our findings emphasize a positive correlation between cognition, assessed both pre-and post-treatment, and motor function; this further confirms that a patient’s motor status is significantly influenced by their cognitive functioning.

These findings are further supported by those of the Norwegian Cognitive Impairment After Stroke (Nor-COAST) prospective multicenter cohort study, which demonstrated that motor performance correlates with global cognition, as well as memory and executive function [19]. Motor function has been also reported to be an important predictor of cognitive impairment after stroke [20]. Given this association, cognitive function has a key role in the planning and execution of rehabilitation efforts and may influence responsiveness to motor rehabilitation interventions. Consequently, cognitive status should be carefully considered when devising targeted treatment strategies for stroke patients [21]. 

Regarding the relationships between functional status and biochemical markers that may influence post-stroke recovery, our findings indicate that pre-rehabilitation MMP-9 serum levels correlate with motor function after rehabilitation, with higher initial MMP-9 levels potentially predicting poorer outcomes in motor function scores assessed by the Activities of Daily Living (ADL) scale. Considering the pleiotropic effects of MMP-9, including its pro-inflammatory impact and blood–brain barrier damage, especially in the acute phase of stroke, this result may reflect lingering effects. Our findings have been corroborated in numerous studies, i.e., that elevated levels of MMP-9, particularly in the acute phase of stroke, are associated with worse outcomes, and a sustained decrease in MMP-9 levels following several days of treatment is indicative of a favorable prognosis [10,22]. 

Indeed, our research was specifically conducted during the subacute phase of stroke; as such, our data may suggest that MMP-9 could play a role in promoting neuroplasticity during this stage. Similarly, an intriguing study by Cai et al. explored the effect of hypoxia response element (HRE)-regulated MMP-9 on glial scars, angiogenesis, and neurogenesis in subacute ischemic stroke. Their findings suggest that MMP-9 plays a beneficial role in the delayed phase of middle cerebral artery occlusion, which is in line with our present findings [23]. They also confirm previous research indicating that MMP-9 may serve as a valuable predictor for cognitive improvement during subacute stroke [5]. Hence, the current study aimed to explore the associations between cognition and the levels of MMP-9, TIMP-1, and MMP-9/TIMP-1 ratio, the latter signifying the active form of MMP-9. It is noteworthy that matrix metalloproteinases typically exist in inactive pro-forms and require conversion to their active structures [24].

The activity of MMPs is intricately regulated by a balance between endogenous inhibitors, such TIMPs, which restrain MMP activity, and endogenous stimulators, like the extracellular matrix metalloproteinase inducer, which promotes MMP activity [25]. Our current results indicate that MMSE score was not significantly related to the levels of MMP-9, TIMP-1, or their ratio; however, notable links were observed between MMSE subdomains and these biochemical parameters. Specifically, a lower initial registration score, also known as immediate recall, was inversely proportional to higher initial total MMP9 and TIMP1. Similarly, changes in this score before and after rehabilitation correlated negatively with changes in the level of active MMP9, as indicated by the MMP9/TIMP1 ratio.

The initial registration subdomain evaluates working memory, which involves the temporary storage of information and engages diffuse cortical processes, particularly the left superior temporal gyrus and left angular gyrus [26]. MMP-9 activity is required for learning and memory, especially in various brain structures and pathways that are subjected to long-term potentiation (LTP) protocols in the hippocampus [6]. However, some studies have reported the presence of MMP-9 also in the cerebral cortex [27]. Interestingly, our data revealed a positive correlation between changes in MMP9 and TIMP1 levels and their association with attention and numeracy values recorded post-therapy. Moreover, the relationship with MMP-9 was also observed among the patients with left-sided paralysis (indicating right-hemisphere stroke). It has been observed that the left hemisphere is more activated compared to the right hemisphere during tasks involving attention, executive processes, and working memory. In contrast, subtraction tasks evoked a greater stimulation of the superior temporal gyrus, inferior frontal gyrus, and thalamus in the right hemisphere [28]. The attention and calculation task in the MMSE involves subtracting the number 7 from 100 five times. Therefore, it is possible that in right-hemisphere stroke patients, ischemia-dependent activation of MMP-9 promotes neuroplasticity.

Significant correlations were also identified in the domain of language functions. It is widely acknowledged that the language-relevant cortex includes Broca’s area (located in the inferior frontal gyrus), Wernicke’s area (situated in the superior temporal gyrus), as well as parts of the middle temporal gyrus, and the inferior parietal and angular gyrus of the parietal lobe in the left hemisphere [28,29]. Our findings show that both the total level of MMP9 and the MMP9/TIMP1 ratio (i.e., indicating the active form of MMP9) are negatively correlated with changes in language domain scores. This may suggest that MMP-9 is not involved in promoting neuroplasticity in language-related areas of the brain, as its involvement is believed to primarily concern hippocampus-dependent learning processes [30]. Furthermore, it is possible that when assessing language function, it is essential to consider potential auditory deficits, which may distort or negatively influence language skills evaluation. This is particularly significant, given human studies suggesting that MMP-9 is involved in neuroplasticity associated with functional responses to cochlear implantation (CI) following the period of auditory deprivation caused by congenital deafness [31]. Additionally, MMP-9 has been identified as a promising prognostic marker for auditory performance after CI. Matusiak et al. concluded that a plasma level of MMP-9 below 150 ng/mL at cochlear implantation indicates a significant chance of a good auditory outcome in an otherwise healthy child [32]. Therefore, it seems to be reasonable to routinely perform hearing tests in stroke populations, which are primarily composed of older individuals who may have underlying hearing deficits, before assessing cognitive function, especially language skills.

It is important to note that our data indicate that improvements in depressive symptoms may also be related to baseline levels of plasma MMP-9, with even more pronounced effects observed for the active form of MMP-9 (MMP-9/TIMP-1 ratio). While previous studies have identified links between MMP-9 and post-stroke depression (PSD), these analyses typically focus on the acute phase of stroke. For instance, Che et al. demonstrated that elevated plasma MMP-9 levels during the acute stage of ischemic stroke were associated with a higher risk of PSD, highlighting the prognostic significance of MMP-9 for PSD [33]. Additionally, strong evidence exists that MMP-9 may influence the development of depression or bipolar depression among individuals without stroke [34,35,36]. Conversely, some studies have reported decreased expression of transcripts and proteins of inter alia MMP-9 in blood samples among individuals with depression [37], but no significant differences in MMP-9 levels between individuals with major depressive disorder and controls have been observed [38]. As such, our data regarding the relationship between improvements in psychological function, as indicated by the GDS scale, and baseline values of total MMP9 and MMP9/TIMP1 are of interest; these findings suggest that delayed ischemia during the subacute phase of a stroke may promote neuroplasticity, leading to improvements in depressive symptoms.

In contrast, no such correlation has been noted in studies examining the acute phase of stroke, during which MMP-9 is known to be primarily involved in processes such as blood–brain barrier damage and neuroinflammation [6]. A more detailed inspection of our results indicates that while baseline MMP-9 levels are related to improvements in depressive symptoms in patients with left-sided paresis (r = −0.81, *p* < 0.0001), this relationship is negligible in those with right-sided paresis (r = −0.20, *p* = 0.40). Moreover, the interaction between the side of paresis and the predictor remains significant even after adjusting for potential confounders such as age and sex. Recent studies do not confirm that stroke lesion laterality may influence the risk and predict post-stroke depression, although this has been demonstrated in the case of post-stroke cognitive impairment (PCI); more specifically, it has been shown that left hemisphere stroke is associated with an increased risk of PCI (42.6% and 53.2% at 6 months and 2 years, respectively) [39]. Currently, there is no clear scientific evidence to explain the phenomenon of correlation between MMP-9 and depression improvement as regards stroke laterality. However, abnormal activity has been observed in brain areas related to language tasks, including the anterior cingulate, the left lateral prefrontal cortex, and the left temporoparietal (Wernicke’s) area in patients with major unipolar depression during a resting state. Interestingly, these abnormalities showed a trend toward normalization, especially in the posterior left temporoparietal area, during treatment [40]. This observation may indirectly suggest the mechanisms responsible for better improvement of depressive symptoms in right hemisphere stroke (evolving left-sided paresis).

### Limitations

While our study has yielded some valuable findings, some limitations should be considered when interpreting them. Firstly, although the sample size is reasonably extensive compared to previous investigations, it is relatively small [41]. Additionally, our participant cohort included both men and women with various long-term conditions (multimorbidity), which may have influenced the results. Furthermore, our study focused specifically on patients with moderate ischemic stroke severity during the subacute phase. Therefore, the findings are characteristic of this defined phase of stroke and level of disability, precluding long-term analyses.

## 4. Materials and Methods

### 4.1. Subjects

The study enrolled a cohort consisting of 40 patients clinically diagnosed with ischemic stroke, admitted to the Department of Neurological Rehabilitation at the Medical University of Lodz between March 2022 and June 2023. The mean age of the participants was 68 ± 10.8. A diagnosis of stroke was established through a meticulous amalgamation of clinical assessments and imaging modalities, adhering rigorously to contemporary stroke criteria. Detailed demographic data are presented in Table 4.

All participants had experienced a moderate cerebrovascular accident, occurring three to four weeks before the study. The exclusion criteria comprised the following: intracerebral hemorrhage, or any serious general condition compounded by respiratory failure severe, or the presence of uncontrolled comorbidities (including neurological disorders such as neurodegenerative diseases, cardiovascular ailments such as recent myocardial infarction, unstable angina pectoris, metabolic or endocrine disorders, or acute infectious diseases).

Post-stroke neurorehabilitation took place over three weeks, excluding weekends; the structure of the rehabilitation program is given in Table 5.

Tailored adjustments were made based on individual physical conditions and progress throughout the rehabilitation process. The program addressed the multifaceted consequences of ischemic stroke, with a specific focus on neurological recovery and functional improvement. The scheduling, duration, and content of each rehabilitative component were tailored to meet the unique requirements and progress of each participant.

### 4.2. Outcome Evaluation

The patients received one of two cognitive assessment tools, depending on their age: the Mini-Mental State Examination (MMSE) or the Montreal Cognitive Assessment (MoCA). The MMSE is a 30-item questionnaire that necessitates vocal responses, comprehension of oral and written commands, spontaneous sentence writing, and interpretation of complex figures. Additionally, it evaluates various cognitive domains including orientation, memory, attention, learning, counting, delayed recall, and structural comprehension. It is widely used as a screening tool for vascular dementia in stroke patients. It is characterized by a sensitivity of 0.96 and a specificity of 0.83, assuming a cut-off point of 23/24 [42]. In contrast, the MoCA test is a concise one-page assessment comprising 30 items. It evaluates various cognitive domains including short-term memory, visuospatial abilities, executive functions, attention and working memory, language proficiency, and orientation in time and space. A score of ≥26 is indicative of normal cognitive function, whereas a score of ≤23 predicts post-stroke cognitive impairment and subsequent functional dependence. This method is extensively employed as a brief cognitive evaluation tool during both the acute/subacute and chronic phases following a stroke [43].

Depressive symptoms were evaluated utilizing one of two standardized assessments depending on patient age: the Geriatric Depression Scale (GDS) or the Beck Depression Inventory (BDI). The GDS is a pragmatic self-report instrument widely employed in clinical settings for assessing depression in older populations. The abbreviated form was chosen for the present study for its established reliability and validity compared to its lengthier counterpart [44]. This version comprises 15 “Yes/No” inquiries. Scores ranging from 0 to 4 typically indicate normal mood states, with the result influenced by age, education level, and underlying health conditions. Conversely, scores of 5–8 indicate mild depression, 9–11 moderate depression, and 12–15 severe depression [45]. The Beck Depression Inventory (BDI) is a widely employed 21-question self-reporting inventory designed to assess the severity of depression in both adolescents and adults. Each question provides respondents with at least four potential answer choices, reflecting the intensity of symptoms, with values ranging from 0 to 3 assigned to each response. The overall score, derived from these questions, determines the severity of depression. Specifically, scores of 0–9 indicate minimal depression, 10–18 mild depression, 19–29 moderate depression, and 30–63 severe depression [46]. 

Physical and motor status were evaluated using the National Institutes of Health Stroke Scale (NIHSS), a tool that is recommended for assessing stroke severity and is known as a significant predictor of post-stroke outcomes [47]. The NIHSS assesses various domains including level of consciousness, eye movements, visual field integrity, facial movements, arm and leg muscle strength, sensation, coordination, language, speech, and neglect. Each domain is assigned a score ranging from 0 to 2, 0 to 3, or 0 to 4. The total score ranges from 0 to 42, with higher scores indicating a more severe stroke.

Finally, independence in activities of daily living (ADL) was determined using the Barthel Index (BI). Its 10 items specifically address self-care and mobility [48]. The total scores can range from 0 to 20, with elevated scores indicating enhanced independence and diminished disability levels. Scores of 17 and above generally denote typical activity levels [49].

### 4.3. Biochemical Assessment

Whole blood samples were obtained in the morning between 7 a.m. and 9 a.m. following an overnight fast; the samples were collected in CPDA1-containing tubes (Sarstedt, Nümbrecht, Germany). Plasma was isolated by differential centrifugation (15 min, 1500× *g*, 25 °C). Sample collection was conducted twice: upon hospital admission and after hospitalization. All procedures related to sample collection and storage were executed in accordance with a predefined protocol.

Total MMP9 level was determined using the Human MMP-9 ELISA Kit (Invitrogen, Waltham, MA, USA), and TIMP1 concentration using the Human TIMP1 ELISA Kit (Abcam, Cambridge, UK). All procedures were performed according to the manufacturer’s protocols. The endpoint reading for analyses was measured at 450 nm, and protein concentration was determined from the standard curve. The level of active MMP9 was calculated based on the MMP9/TIMP1 ratio. All analytical procedures were conducted in accordance with the prescribed methodologies.

### 4.4. Statistical Analysis

Nominal variables were described as number with frequency. Continuous variables were subjected to the Shapiro–Wilk test to determine their distribution; based on the result, the data were given as median with 1st to 3rd quartile (non-normal distribution) or mean with standard deviation (normal distribution). Post-rehabilitation outcomes were compared with pre-rehabilitation measures using Student’s *t*-test. The association between two variables of interest was evaluated with Pearson’s r. Covariate-adjusted models of association and bivariate interaction testing were performed with the use of general linear modeling. *p*-values below 0.05 were considered statistically significant. The analysis was performed with STATISTICA 13.3 software (StatSoft; Tulsa, OK, USA).

## 5. Conclusions

Although the subacute phase of stroke is characterized by the gradual attenuation of neuroinflammation and tissue degradation, together with the intensification of neuroplasticity and angiogenesis processes, few studies have examined the recovery trajectories in this period. Thus, it is pertinent to investigate the potential role of MMP-9 in facilitating post-stroke recovery during this critical phase. Our study suggests that MMP-9 can positively impact psychological functions, such as alleviating depressive symptoms and enhancing specific cognitive, including calculation. While our findings do not indicate a significant correlation between total MMSE score and the levels of MMP9, TIMP1, or their ratio, there is a need for further exploration in this area. 

We propose that the distinct MMP-9 pathways within different brain regions may underlie diverse cognitive domains, necessitating further separate analyses. Notably, Zhao et al. demonstrated a negative relationship between serum MMP-9 levels and the total score in MoCA, as well as in specific subdomains including visual-spatial executive function, naming, attention, language, and delayed recall [50]. Our present study validates previous findings that serum MMP-9 levels before rehabilitation are negatively correlated with motor function after rehabilitation. 

Our data reveal ambiguous trends regarding changes in MMP9 and TIMP1 level or MMP9/ TIMP1 during post-stroke rehabilitation and their implications for functional status, as well as their influence on cognition and depression recovery in stroke patients. As such, further research with larger sample sizes is warranted.

Finally, a positive correlation was found between cognition and motor function. This underscores the necessity of considering both aspects when formulating rehabilitation strategies.

## Figures and Tables

**Table 1:** Correlation analysis of cognitive and psychological function parameters with biochemical parameters.

		MMP9 Baseline Value (ng/mL)	Delta MMP9 (log%)	TIMP1 Baseline Value (ng/mL)	MMP9/TIMP1 Baseline Value	Delta MMP9/TIMP1 (log%)
MMSE total	Before rehabilitation	r = −0.00, *p* = 0.999	r = −0.11, *p* = 0.490	r = −0.13, *p* = 0.439	r = 0.06, *p* = 0.726	r = −0.19, *p* = 0.253
	After rehabilitation	r = −0.10, *p* = 0.564	r = 0.01, *p* = 0.955	r = −0.16, *p* = 0.319	r = −0.03, *p* = 0.852	r = −0.08, *p* = 0.633
	Delta value	r = −0.17, *p* = 0.312	r = 0.26, *p* = 0.108	r = −0.01, *p* = 0.937	r = −0.18, *p* = 0.277	r = 0.26, *p* = 0.104
Registration	Before rehabilitation	r = −0.41, *p* = 0.009	r = 0.22, *p* = 0.173	r = −0.39, *p* = 0.015	r = 0.03, *p* = 0.851	r = 0.00, *p* = 0.998
	Delta value	r = 0.06, *p* = 0.720	r = −0.36, *p* = 0.026	r = −0.15, *p* = 0.348	r = 0.11, *p* = 0.497	r = −0.33, *p* = 0.041
Attention and Calculation	Delta value	r = 0.01, *p* = 0.943	r = 0.43, *p* = 0.006	r = −0.00, *p* = 0.987	r = 0.06, *p* = 0.737	r = 0.37, *p* = 0.019
Language	Delta value	r = −0.46, *p* = 0.003	r = 0.13, *p* = 0.426	r = −0.15, *p* = 0.346	r = −0.38, *p* = 0.017	r = 0.06, *p* = 0.729

**Table 2:** Correlation analysis between cognitive and psychological function parameters and biochemical parameters in patients with left-sided paresis.

		MMP9 Baseline Value (ng/mL)	Delta MMP9 (log%)	TIMP1 Baseline Value (ng/mL)	Delta TIMP1 (log%)	MMP9/TIMP1 Baseline Value	Delta MMP9/TIMP1 (log%)
Attention and Calculation	Before rehabilitation	r = −0.40, *p* = 0.077	r = 0.08, *p* = 0.734	r = −0.24, *p* = 0.300	r = 0.26, *p* = 0.271	r = −0.10, *p* = 0.690	r = −0.08, *p* = 0.745
	After rehabilitation	r = −0.47, *p* = 0.035	r = 0.45, *p* = 0.047	r = −0.31, *p* = 0.180	r = 0.39, *p* = 0.091	r = −0.04, *p* = 0.858	r = 0.15, *p* = 0.514
	Delta value	r = 0.06, *p* = 0.802	r = 0.41, *p* = 0.069	r = 0, *p* = 0.984	r = 0.06, *p* = 0.792	r = 0.10, *p* = 0.685	r = 0.31, *p* = 0.188
Language	Before rehabilitation	r = 0.26, *p* = 0.277	r = −0.23, *p* = 0.323	r = −0.08, *p* = 0.730	r = 0.23, *p* = 0.324	r = 0.50, *p* = 0.025	r = −0.32, *p* = 0.165
	After rehabilitation	r = −0.23, *p* = 0.337	r = −0.18, *p* = 0.443	r = −0.42, *p* = 0.064	r = 0.51, *p* = 0.021	r = 0.16, *p* = 0.500	r = −0.44, *p* = 0.054
	Delta value	r = −0.45, *p* = 0.048	r = 0.19, *p* = 0.412	r = −0.13, *p* = 0.589	r = −0.01, *p* = 0.961	r = −0.54, *p* = 0.013	r = 0.17, *p* = 0.482

**Table 3:** Correlation analysis between cognitive and psychological function parameters and biochemical parameters in patients with right-sided paresis.

		MMP9 Baseline Value (ng/mL)	Delta MMP9 (log%)	TIMP1 Baseline Value (ng/mL)	Delta TIMP1 (log%)	MMP9/TIMP1 Baseline Value	Delta MMP9/TIMP1 (log%)
Language	Before rehabilitation	r = 0.15, *p* = 0.535	r = 0.07, *p* = 0.781	r = 0.17, *p* = 0.487	r = −0.36, *p* = 0.129	r = 0.10, *p* = 0.671	r = 0.30, *p* = 0.207
	After rehabilitation	r = −0.12, *p* = 0.612	r = 0.09, *p* = 0.724	r = 0.10, *p* = 0.690	r = −0.28, *p* = 0.244	r = −0.07, *p* = 0.780	r = 0.27, *p* = 0.265
	Delta value	r = −0.47, *p* = 0.044	r = 0.02, *p* = 0.947	r = −0.15, *p* = 0.548	r = 0.20, *p* = 0.419	r = −0.29, *p* = 0.221	r = −0.11, *p* = 0.647

**Table 4:** Demographic characteristics.

Parameter	Mean (SD) or Number (Frequency)
Sociodemographic	
Sex—female	14 (35%)
Sex—male	26 (65%)
Age (years)	68 (10.8)
Comorbidity and treatment	
Hypertension	24 (60%)
Diabetes	11 (28%)
Atherosclerosis	7 (18%)
Thrombolytic treatment	5 (13%)
Blood parameters	
Sodium	138.9 (2.87)
Potassium	4.3 (0.36)
WBC	7.55 (1.96)
RBC	4.41 (0.57)
Hb	13.31 (1.54)
HCT	40.8 (4.26)
Urea	32.8 (15.1)
Creatinine	0.82 (0.24)

**Table 5:** The rehabilitation program.

Methods	Description
Neurophysiological session (morning)	A 30 min session focused on implementing techniques derived from daily activities, along with an additional 30 min dedicated to repetitive task exercises or balance exercises
Psychotherapy	A 15 min session designated for psychotherapeutic interventions
Aerobic training	Aerobic exercise sessions were conducted 2–3 times daily, each one lasting 10 min, with intervals spaced at 60 min intervals

## Data Availability

Data is contained within the article and Supplementary Materials.

## Supplementary Materials

The following supporting information can be downloaded at: https://www.mdpi.com/article/10.3390/ijms25115745/s1.
