# Supplementary material for: Exploring the Role of MMP-9 and MMP-9/TIMP-1 Ratio in Subacute Stroke Recovery: A Prospective Observational Study
Source: Int J Mol Sci. 2024 May 25;25(11):5745. doi: 10.3390/ijms25115745 (PMC11172289; doi:10.3390/ijms25115745)
Supplement: Supplementary file 1 [file ijms-25-05745-s001.zip › Table S1.pdf]

Table S1. Correlation analysis of cognitive and psychological function parameters with biochemical parameters

		MMP9 baseline value [ng/mL]	Delta MMP9 log%]	TIMP1 baseline value [ng/mL]	Delta TIMP1 [log%]	MMP9/TIMP1 baseline value	Delta MMP9/TIMP1 [log%]
MMSE total	Before rehabilitation	-0.00, p=.999	-0.11, p=.490	-0.13, p=.439	0.15, p=.378	0.06, p=.726	-0.19, p=.253
	After rehabilitation	-0.10, p=.564	0.01, p=.955	-0.16, p=.319	0.15, p=.376	-0.03, p=.852	-0.08, p=.633
	Delta value	-0.17, p=.312	0.26, p=.108	-0.01, p=.937	-0.06, p=.733	-0.18, p=.277	0.26, p=.104
Orientation	Before rehabilitation	0.08, p=.621	-0.22, p=.172	-0.12, p=.473	0.10, p=.536	0.10, p=.539	-0.26, p=.113
	After rehabilitation	0.16, p=.317	-0.30, p=.062	-0.04, p=.801	0.07, p=.682	0.15, p=.356	-0.31, p=.057
	Delta value	0.12, p=.476	-0.08, p=.632	0.15, p=.353	-0.08, p=.630	0.06, p=.717	-0.02, p=.893
Registration	Before rehabilitation	-0.41, p=.009	0.22, p=.173	-0.39, p=.015	0.33, p=.041	0.03, p=.851	0.00, p=.998
	After rehabilitation	-0.14, p=.408	-0.26, p=.112	-0.34, p=.033	0.18, p=.275	0.13, p=.432	-0.34, p=.037
	Delta value	0.06, p=.720	-0.36, p=.026	-0.15, p=.348	0.02, p=.893	0.11, p=.497	-0.33, p=.041
Attention and Calculation	Before rehabilitation	-0.23, p=.167	-0.04, p=.799	-0.15, p=.355	0.21, p=.210	-0.16, p=.336	-0.16, p=.330
	After rehabilitation	-0.26, p=.106	0.29, p=.070	-0.19, p=.257	0.26, p=.112	-0.15, p=.373	0.10, p=.528
	Delta value	0.01, p=.943	0.43, p=.006	-0.00, p=.987	0.01, p=.937	0.06, p=.737	0.37, p=.019
Recall	Before rehabilitation	-0.07, p=.655	0.15, p=.362	0.06, p=.713	-0.05, p=.780	-0.20, p=.233	0.16, p=.330
	After rehabilitation	0.12, p=.474	0.20, p=.218	0.05, p=.745	-0.12, p=.483	-0.05, p=.755	0.25, p=.129
	Delta value	0.19, p=.242	0.08, p=.650	0.00, p=.995	-0.08, p=.630	0.12, p=.451	0.11, p=.490
Language	Before rehabilitation	0.18, p=.264	-0.09, p=.583	0.05, p=.767	-0.02, p=.887	0.21, p=.211	-0.07, p=.690
	After rehabilitation	-0.19, p=.247	0, p=.993	-0.08, p=.626	0.06, p=.713	-0.08, p=.621	-0.04, p=.832
	Delta value	-0.46, p=.003	0.13, p=.426	-0.15, p=.346	0.10, p=.553	-0.38, p=.017	0.06, p=.729
Constructional Praxis	Before rehabilitation	-0.03, p=.837	0.23, p=.151	-0.21, p=.196	0.08, p=.622	0.06, p=.733	0.16, p=.335
	After rehabilitation	0.13, p=.435	0.1, p=.547	0.12, p=.484	-0.25, p=.119	-0.06, p=.722	0.24, p=.142
	Delta value	0.15, p=.363	-0.13, p=.424	0.31, p=.058	-0.31, p=.056	-0.11, p=.516	0.07, p=.679
GDS	Before rehabilitation	0.26, p=.103	-0.22, p=.181	0.00, p=.980	0.13, p=.423	0.31, p=.052	-0.27, p=.094
	After rehabilitation	0.05, p=.775	-0.33, p=.041	0.01, p=.936	0.02, p=.927	0.06, p=.720	-0.30, p=.064
	Delta value	-0.36, p=.025	-0.13, p=.439	0.01, p=.940	-0.19, p=.245	-0.42, p=.008	0.00, p=.994
