# Supplementary material for: Exploring the Role of MMP-9 and MMP-9/TIMP-1 Ratio in Subacute Stroke Recovery: A Prospective Observational Study
Source: Int J Mol Sci. 2024 May 25;25(11):5745. doi: 10.3390/ijms25115745 (PMC11172289; doi:10.3390/ijms25115745)
Supplement: Supplementary file 1 [file ijms-25-05745-s001.zip › Table S2.pdf]

Table S2. Correlation analysis of cognitive and psychological function parameters with biochemical parameters with patients with left-sided paresis.

		MMP9 baseline value [ng/mL]	Delta MMP9 log%]	TIMP1 baseline value [ng/mL]	Delta TIMP1 [log%]	MMP9/TIMP1 baseline value	Delta MMP9/TIMP1 [log%]
MMSE total	Before rehabilitation	-0.07, p=.782	-0.17, p=.471	-0.39, p=.088	0.42, p=.063	0.40, p=.083	-0.38, p=.101
	After rehabilitation	-0.10, p=.686	-0.04, p=.862	-0.49, p=.029	0.43, p=.060	0.40, p=.082	-0.27, p=.243
	Delta value	-0.02, p=.934	0.30, p=.202	0.02, p=.933	-0.19, p=.422	-0.18, p=.435	0.35, p=.127
Orientation	Before rehabilitation	0.17, p=.487	-0.36, p=.122	-0.39, p=.089	0.43, p=.058	0.48, p=.034	-0.54, p=.015
	After rehabilitation	0.35, p=.133	-0.42, p=.067	-0.20, p=.409	0.30, p=.195	0.55, p=.012	-0.51, p=.020
	Delta value	0.38, p=.097	-0.12, p=.607	0.41, p=.069	-0.27, p=.243	0.15, p=.526	0.05, p=.828
Registration	Before rehabilitation	NA	NA	NA	NA	NA	NA
	After rehabilitation	-0.02, p=.924	-0.24, p=.305	-0.56, p=.011	0.30, p=.204	0.26, p=.267	-0.37, p=.113
	Delta value	-0.02, p=.924	-0.24, p=.305	-0.56, p=.011	0.30, p=.204	0.26, p=.267	-0.37, p=.113
Attention and Calculation	Before rehabilitation	-0.40, p=.077	0.08, p=.734	-0.24, p=.300	0.26, p=.271	-0.10, p=.690	-0.08, p=.745
	After rehabilitation	-0.47, p=.035	0.45, p=.047	-0.31, p=.180	0.39, p=.091	-0.04, p=.858	0.15, p=.514
	Delta value	0.06, p=.802	0.41, p=.069	0, p=.984	0.06, p=.792	0.10, p=.685	0.31, p=.188
Recall	Before rehabilitation	-0.35, p=.128	0.29, p=.215	-0.04, p=.865	-0.18, p=.446	-0.31, p=.179	0.34, p=.142
	After rehabilitation	-0.31, p=.185	0.35, p=.125	-0.14, p=.547	-0.27, p=.243	-0.17, p=.462	0.45, p=.049
	Delta value	0.04, p=.862	0.09, p=.713	-0.13, p=.597	-0.12, p=.618	0.16, p=.505	0.14, p=.559
Language	Before rehabilitation	0.26, p=.277	-0.23, p=.323	-0.08, p=.730	0.23, p=.324	0.50, p=.025	-0.32, p=.165
	After rehabilitation	-0.23, p=.337	-0.18, p=.443	-0.42, p=.064	0.51, p=.021	0.16, p=.500	-0.44, p=.054
	Delta value	-0.45, p=.048	0.19, p=.412	-0.13, p=.589	-0.01, p=.961	-0.54, p=.013	0.17, p=.482
Constructional Praxis	Before rehabilitation	-0.38, p=.100	0.4, p=.081	-0.37, p=.106	0.10, p=.671	0.01, p=.977	0.27, p=.243
	After rehabilitation	0.23, p=.331	0.17, p=.477	0.04, p=.865	-0.29, p=.218	0.18, p=.459	0.30, p=.198
	Delta value	0.56, p=.011	-0.16, p=.502	0.35, p=.125	-0.39, p=.093	0.18, p=.454	0.08, p=.725
GDS	Before rehabilitation	0.43, p=.059	-0.4, p=.079	0.13, p=.591	0.30, p=.202	0.40, p=.081	-0.50, p=.025
	After rehabilitation	0.16, p=.512	-0.47, p=.037	0.27, p=.243	0.10, p=.687	-0.1, p=.685	-0.44, p=.052
	Delta value	-0.46, p=.039	-0.08, p=.749	0.22, p=.347	-0.34, p=.141	-0.81, p=.000	0.13, p=.592
