# Supplementary material for: Exploring the Role of MMP-9 and MMP-9/TIMP-1 Ratio in Subacute Stroke Recovery: A Prospective Observational Study
Source: Int J Mol Sci. 2024 May 25;25(11):5745. doi: 10.3390/ijms25115745 (PMC11172289; doi:10.3390/ijms25115745)
Supplement: Supplementary file 1 [file ijms-25-05745-s001.zip › Table S3.pdf]

Table S3. Correlation analysis of cognitive and psychological function parameters with biochemical parameters with patients with right-sided paresis.

		MMP9 baseline value [ng/mL]	Delta MMP9 log%]	TIMP1 baseline value [ng/mL]	Delta TIMP1 [log%]	MMP9/TIMP1 baseline value	Delta MMP9/TIMP1 [log%]
MMSE total	Before rehabilitation	0.13, p=.604	-0.06, p=.814	0.14, p=.554	-0.30, p=.204	-0.06, p=.798	0.14, p=.565
	After rehabilitation	-0.03, p=.917	0.05, p=.826	0.15, p=.539	-0.30, p=.216	-0.19, p=.442	0.25, p=.307
	Delta value	-0.31, p=.202	0.21, p=.381	-0.04, p=.876	0.11, p=.642	-0.19, p=.425	0.14, p=.575
Orientation	Before rehabilitation	0.01, p=.955	-0.04, p=.882	0.14, p=.563	-0.39, p=.097	-0.09, p=.700	0.22, p=.368
	After rehabilitation	-0.11, p=.661	-0.07, p=.769	0.14, p=.559	-0.4, p=.090	-0.17, p=.495	0.19, p=.439
	Delta value	-0.15, p=.552	-0.02, p=.933	-0.08, p=.760	0.20, p=.406	-0.03, p=.900	-0.15, p=.533
Registration	Before rehabilitation	-0.52, p=.023	0.34, p=.152	-0.47, p=.042	0.48, p=.039	0.09, p=.710	0.03, p=.908
	After rehabilitation	-0.21, p=.385	-0.29, p=.232	-0.18, p=.458	0.04, p=.883	0.10, p=.687	-0.31, p=.197
	Delta value	0.11, p=.658	-0.48, p=.037	0.11, p=.656	-0.25, p=.297	0.04, p=.871	-0.31, p=.192
Attention and Calculation	Before rehabilitation	0.00, p=.995	-0.22, p=.359	-0.03, p=.915	0.06, p=.814	-0.13, p=.588	-0.26, p=.284
	After rehabilitation	-0.07, p=.781	0.13, p=.590	-0.07, p=.771	0.09, p=.724	-0.16, p=.511	0.07, p=.762
	Delta value	-0.09, p=.706	0.49, p=.032	-0.06, p=.816	0.03, p=.895	-0.02, p=.923	0.47, p=.043
Recall	Before rehabilitation	0.20, p=.411	-0.05, p=.851	0.15, p=.536	0.17, p=.495	-0.17, p=.480	-0.15, p=.527
	After rehabilitation	0.52, p=.023	0.02, p=.923	0.24, p=.332	0.01, p=.955	0.04, p=.857	0.01, p=.953
	Delta value	0.35, p=.143	0.06, p=.816	0.11, p=.644	-0.11, p=.652	0.17, p=.485	0.13, p=.599
Language	Before rehabilitation	0.15, p=.535	0.07, p=.781	0.17, p=.487	-0.36, p=.129	0.10, p=.671	0.30, p=.207
	After rehabilitation	-0.12, p=.612	0.09, p=.724	0.10, p=.690	-0.28, p=.244	-0.07, p=.780	0.27, p=.265
	Delta value	-0.47, p=.044	0.02, p=.947	-0.15, p=.548	0.20, p=.419	-0.29, p=.221	-0.11, p=.647
Constructional Praxis	Before rehabilitation	0.27, p=.266	0.04, p=.875	-0.09, p=.723	0.06, p=.821	0.09, p=.707	0.00, p=.993
	After rehabilitation	-0.02, p=.932	-0.07, p=.769	0.30, p=.215	-0.21, p=.387	-0.37, p=.119	0.07, p=.790
	Delta value	-0.31, p=.192	-0.09, p=.722	0.28, p=.246	-0.19, p=.433	-0.33, p=.168	0.04, p=.879
GDS	Before rehabilitation	0.10, p=.698	-0.01, p=.972	-0.12, p=.615	0, p=.989	0.26, p=.282	-0.01, p=.965
	After rehabilitation	-0.04, p=.858	-0.16, p=.510	-0.20, p=.420	-0.09, p=.705	0.15, p=.529	-0.10, p=.686
	Delta value	-0.22, p=.362	-0.22, p=.368	-0.08, p=.743	-0.14, p=.567	-0.21, p=.395	-0.13, p=.607
